# Defining the transition from new to normal: a qualitative investigation of the clinical change process

**DOI:** 10.21203/rs.3.rs-4366064/v1

**Published:** 2024-05-21

**Authors:** Santana R. Silver, Kayla Christine Jones, Kimberly Hook, Erika L. Crable, Emily R. George, Janet R. Serwint, Kirsten Austad, Allan Walkey, Mari-Lynn Drainoni

**Affiliations:** Boston University Chobanian & Avedisian School of Medicine; Boston University Chobanian & Avedisian School of Medicine; Harvard T.H. Chan School of Public Health; University of California, San Diego; Boston University School of Public Health; Johns Hopkins Medicine; Boston University Chobanian & Avedisian School of Medicine; UMass Chan School of Medicine; Boston University Chobanian & Avedisian School of Medicine

**Keywords:** Clinical practice change, normalization, sustainment, evidence-based practice, healthcare delivery, implementation science

## Abstract

**Background:**

Understanding how and when a new evidence-based clinical intervention becomes standard practice is crucial to ensure that healthcare is delivered in alignment with the most up-to-date knowledge. However, rigorous methods are needed to determine when a new clinical practice becomes normalized to the standard of care. To address this gap, this study qualitatively explores how, when, and why a clinical practice change becomes normalized within healthcare organizations.

**Methods:**

We used purposive sampling to recruit clinical leaders who worked in implementation science across diverse health contexts. Enrolled participants completed semi-structured interviews. Qualitative data analysis was guided by a modified version of the Normalization Process Theory (NPT) framework to identify salient themes. Identified normalization strategies were mapped to the Expert Recommendations for Implementation Change (ERIC) project.

**Results:**

A total of 17 individuals were interviewed. Participants described four key signals for identifying when a novel clinical practice becomes the new normal: 1) integration into existing workflows; 2) scaling across the entire organizational unit; 3) staff buy-in and ownership; and 4) sustainment without ongoing monitoring. Participants identified salient strategies to normalize new clinical interventions: 1) taking a patient approach; 2) gaining staff buy-in and ownership; and 3) conducting ongoing measurement of progress towards normalization.

**Conclusions:**

The results offer valuable insight into the indicators that signify when a novel clinical practice becomes normalized, and the strategies employed to facilitate this transition. These findings can inform future research to develop instruments that implementation leaders can use to systematically measure the clinical change process.

## BACKGROUND

Understanding how and when a new evidence-based clinical intervention becomes standard practice is crucial to ensure that healthcare is effectively and efficiently delivered in alignment with the most up-to-date knowledge. This is not the current reality; there is an estimated 17-year time lag from when new evidence is published to the time it is implemented into routine practice.^1^ Diverse implementation barriers contribute to this research-to-practice gap, including the lack of intraorganizational dedicated efforts to rigorously investigate how, when, and why a new evidence-based practice (EBP) has been institutionalized.^2–6^

Clinical practice changes often transform workflows, communication networks, staff responsibilities, and ultimately how healthcare is delivered.^7–10^ Adopting new clinical practices disturbs the status quo and requires staff to learn and unlearn behaviors.^11,12^ These multiple implementation challenges are exacerbated by the additional barriers of limited resources, structural obstacles, and differing staff values and beliefs.^13^ Additionally, patient and family factors (e.g., historical medical mistrust or mistreatment, inaccuracies in web-based information, cultural norms, etc.) may also delay or otherwise hinder implementation efforts. To overcome implementation barriers to practice changes, healthcare systems must allocate monetary and non-monetary resources (e.g., time, staffing, implementation taskforces) and tailored communications.^14–16^

However, without rigorous methods to determine when an implementation effort focused on EBP adoption (i.e., clinical practice change) is no longer needed because the EBP has evolved to the standard of care in that setting, healthcare systems run the risk of continuing redundant implementation efforts, unnecessarily using precious resources, and causing change fatigue among staff. While quantitative measures exist to predict and enhance program *sustainment*,^17,18^ these tools stop at sustainability and do not capture predictors for the next stage of true program *normalization* within healthcare settings. Identifying indicators to predict when a practice change transitions from *active sustainment* (when the practice is still perceived as new and requires reminders to sustain) to *normalization* (when the practice is perceived as standard and no longer requires reminders to sustain) is crucial to fostering and evaluating successful implementation efforts that result in the normalization of care practice changes. Additionally, a systematic way to identify when new evidence has been incorporated into standard practice will enhance the ability to track local research-to-practice timelines.

In order to begin to define how and when a practice change transitions from new to normal, we sought to explore strategies that clinical leaders use to facilitate change and how leaders define when successful practice change has occurred. We conducted in-depth qualitative interviews with clinical and quality improvement leaders in healthcare settings across the US in order to investigate how new evidence-based practices, guidelines, or approaches to clinical medicine move beyond initial uptake and progress to normalization. Though literature exists on effective implementation and sustainment strategies,^19–22^ there is a research gap on strategies used to move a new clinical practice beyond sustainment and to internalized normalization within a healthcare setting. Therefore, this study seeks to address that gap by exploring and classifying normalization strategies employed by healthcare leaders.

## METHODS

### Participant Recruitment

We used a purposive sampling technique to recruit individuals who were identified as clinical leaders in implementation or quality improvement within diverse health contexts. Individuals were initially identified via the study team and screened based upon their previous professional contributions. Snowball sampling was then used to recruit additional subjects: at the end of each interview participants were asked to identify peers to participate. Participants provided only publicly available information (i.e., names of nominees), which we then used to locate contact information on university or health system websites. The study team contacted potential participants by email, inviting them to participate in an individual, semi-structured interview. Interviewers met with potential participants via Zoom to explain the study and confirm eligibility. To be eligible, candidates had to speak English, be employed by a health setting (academic medical center, hospital, or community health center), and be considered a leader in implementation or quality improvement (e.g., chief quality officer, director of quality and safety, etc.). Eligible candidates gave verbal consent before interviews were conducted. The study was approved by the Boston Medical Center and Boston University Medical Campus Institutional Review Board.

### Data Collection

A semi-structured interview guide was developed by the study team. We asked four open-ended questions with probes to gain participant insight into the process of how and when an intervention is fully transitioned to standard practice. Specifically, we asked participants: 1) what healthcare practice change means to them; 2) how change relates to sustainability; 3) how they know when sustainability has been achieved; and 4) to describe healthcare practice change examples they had encountered at their institutions to understand the process from initiation to normalization. Two members of the study team with experience in qualitative research (KH and EG) conducted interviews between March and May 2021. Interviews were conducted over Zoom, audio-recorded, and transcribed verbatim by a professional transcription company. Participants were not compensated for their time.

### Data Analysis

Qualitative data analysis was guided by a modified version of the Normalization Process Theory (NPT) framework to identify salient themes. NPT is a theoretical framework used to investigate how interventions become embedded and sustained in social contexts. NPT conceptualizes an intervention as an ensemble of beliefs, behaviors, and acts.^23–25^ The NPT coding manual developed by May et al.^24^ translates the theoretical constructs into a qualitative analytic codebook consisting of three domains: contexts, mechanisms, and outcomes (See Supplemental Materials).

Data analysis was conducted by four analysts (SS, KCJ, RS, GCR) with qualitative coding and analytic experience. All members of the analysis team applied the draft codebook to six initial interview transcripts. The team met to review and reconcile coding discrepancies, revising the codebook based on team consensus. After high inter-coder agreement was achieved on the sixth transcript, the codebook was finalized and applied to double code the remaining transcripts using the NVivo 12.0 software program.^26^ After coding was complete, each member of the data analysis team independently reviewed coded data to develop initial themes. The analysts then met to compare and discuss preliminary themes, organizing them into two emergent categories including normalization signals and strategies. Specifically, themes related to 1) indicators of the change process (i.e., any measured or immeasurable signal that an implementation effort was no longer needed because the focal EBP was normalized as the standard of care); and 2) normalization strategies utilized during this change process.

The lead analysist (SS) finalized consensus themes based on team feedback. During this finalization stage, she mapped each identified normalization strategy to relevant implementation strategies defined and sorted by the Expert Recommendations for Implementing Change (ERIC) project.^20,27^ Briefly, the ERIC project developed a compilation of implementation strategy terms and definitions based on input from implementation science experts. We matched normalization strategies evident in our qualitative data to ERIC strategies in order to identify relationships between normalization and implementation strategies while enhancing the conceptual clarity, consistency, and relevance of our findings to both the field of implementation science and clinical practice.

## RESULTS

Study participants: Seventeen individuals at different healthcare settings across the United States participated in the qualitative interviews (see Table 1). The majority of the sample was female (*n* = 14) and had a master’s and/or doctoral degree (*n* = 15). The median age range was 46–55 years old and the mean number of years in healthcare practice was 22. All healthcare institutions were in an urban setting, but also served rural populations, particularly those located in the Midwest (*n* = 4), Southeast (*n* = 3), and South (n = 1) regions. The majority of participating health settings were academic medical centers (*n* = 14) and considered large in terms of in-patient size (*n* = 10). Interview length ranged from 45–75 minutes.

### Qualitative themes:

**Figure 1:**
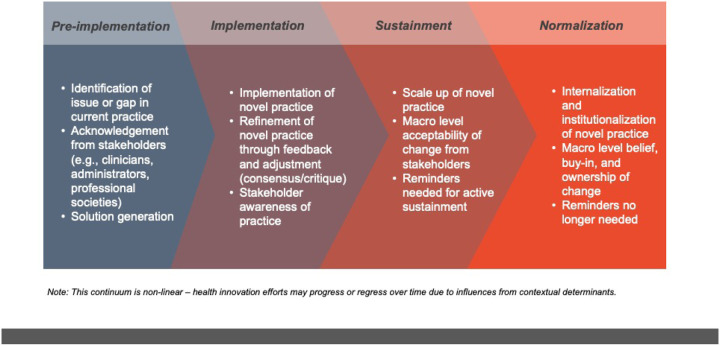
Legend not included with this version.

We identified two categories of themes: 1) themes regarding signals of when a new clinical practice is considered to be normalized within clinical care; and 2) themes related to strategies about how to normalize new clinical innovations. Participants described four key signals for identifying when a novel clinical practice becomes the new normal: 1) integrated seamlessly into existing workflows; 2) scaled across the entire organizational unit; 3) has strong staff buy-in and ownership; and 4) no longer needs monitoring and evaluation to be sustained. Themes related to strategies to normalize new clinical interventions included: a) taking a patient approach that starts slow and gains momentum over time; b) identifying and using methods to gain staff buy-in and ownership; and c) conducting ongoing measurement of progress towards a lasting organizational change. Both types of themes – normalization signals and strategies – are described in detail below. Based on these themes, Fig. 1 illustrates the non-linear process to normalization of a novel practice, comprised of the familiar continuum of pre-implementation, implementation, active sustainment, and ultimately normalization.

#### 1. Signals of when a new, evidence-based clinical practice is normalized in clinical care:

**a. New clinical practices become normalized when they are** integrated seamlessly into existing workflows. A major emergent theme from the qualitative interviews was that a new intervention cannot transition to standard practice until it is seamlessly integrated into existing workflows. When changes are fully hardwired into current procedures and systems, new practices are more likely to become the default in daily care.

“[Implementation leaders] really tried to make sure [the practice change] was integrated into our workflows. So the ways that our nurses practice now, I bet if I were on the floor right now and asked them, “Hey, could you live without x?” They’d be like, “Oh my God, don’t take that away.” ‘Cause it changed the way that they practice medicine.”

(203)

Qualitative data revealed that integration of new practices into existing workflow facilitates the change process by making work tasks easier for staff, thereby also fostering staff buy-in of the change. A participant describes the way in which defaulting workflows to the EBP makes the transition easier for staff:

“I think about getting people bought into the idea that something different is possible and that either doing things a different way or standardizing our practice in some way is a bene fit…that means making it the easier thing to do. Like figuring out ways to default workflows so that doing the right thing is the easy way to do it”

(207)

This participant provides an example of using the EHR to default workflows by adding an order set to prompt physicians to adhere to the practice change (i.e., setting a sedation score depending on how sick pediatric patients are in order to titrate medication).

“The practice change was that we put an order in, but I think the reason it became sustainable over time is because the way that we titrate our medications is based on that score. So you can’t do the second part of your job without doing the first part, if that makes sense.”

(207)

**b. Novel clinical practices become normalized when they are** scaled across the entire practice setting. Participants also agreed that achieving scale, or reach, of the intervention is necessary for standardization of the practice change to occur. Many participants referred to this standardization process as “culture change”. To become normalized, the practice change has to be widespread across the practice setting, which can vary from a single department (i.e., pediatrics) to an entire hospital system depending on the type of EBP.

Culture change in healthcare is taking something that has been in place and literally revamping it across the organization, not just in one department or one entity or even in one group.”

(203)

Participants identified an effective scaling approach as initially implementing the practice on a small and local level and eventually expanding it within the organization. For instance, a participant describes the way in which changes to investigation of safety events were introduced slowly and trialed in one unit before scaling across the institution.

“One of the first things we tried to work on was, how do we integrate the frontline folks who were actually involved in an error into the investigation…We need that cultural change to happen locally before people will start reporting more. And so it was successful in the end, and the way it spread was as it started getting brought up at leadership meetings, other areas started to hear that and said I want to do something like that.”

(200)

**c. Novel clinical practices become normalized when there is** staff buy-in and ownership. Another major component to gauge when a new practice becomes standard is the level of staff buy-in and ownership over the change. Participants emphasized that staff buy-in and ownership are necessary to move the practice change from a new to routine practice.

“Those working in that microsystem or macrosystem are at the heart of the change. The change is happening within them or within their microsystem. Therefore, they have to ultimately own it.”

(113)

For this particular example, the participant detailed a practice change that included developing an accountable care team in each hospital unit in order to efficiently respond to problems at the microsystems level. According to the participant, this structure change enabled individual units to solve the systemwide issue of delayed hospital discharge. The change was made possible by staff buy-in, as staff recognized the value and were empowered to solve problems:

“What it did was it flips the power pyramid upside down. What never will work is top-down solutions. So a bunch of senior leaders who don’t actually work on the front lines, instructing people how to solve their department efficiency problem…Equip them to do that, and then they themselves implement the system themselves.”

(113)

Related to the aforementioned requirement of scaled change, participants noted that the staff buy-in must also be widespread.

“I’ve got complete buy-in from the person who washes the dishes to the CEO of the hospital. Everybody is moving in the same direction.”

(203)

**d. Novel clinical practices become normalized when they are** continuously implemented without requiring ongoing monitoring. The last major signal of when a new practice change is officially standardized into routine care (i.e., ‘culture change’) is when the practice is sustained even after incentives, rewards, reminders (i.e., what several participants referred to as “guardrails”) are removed. In this sense, the transition to true normalized practice change is when the EBP is internalized by staff and moves beyond time-limited behavior change that can be sustained externally.

“Some issues may need reminders like, ‘Oh, remember, we’re still doing this all the time, even though it’s not on our Quality Dashboard anymore’ versus [culture change] is what we think is best practice for pediatrics and this is going to improve child health and this is the evidence behind it. Sustainability is still -- there’s some guardrails in place, and most culture changes are more of an internal sense of, this is what we do, or this is just normal for us.”

(199)

Participants said the reason that true adoption and internalization of a standard practice do not need the same “guardrails” as mere behavior change is because it is driven by a shift in beliefs and values.

“Culture change, I think it being something a little bit more global, it brings in things that go beyond the actual behavior. The behavior is sustainable. Whereas culture is a more multidimensional construct in which we’re thinking about the attitudes, and beliefs since, and intentions, and principles, and priorities that are associated. Cultural change would be more about setting as a mission and a priority.”

(140)

As a result, implementation leaders can test progress towards true standardization of a new intervention by removing the guardrails and checking to see whether the practice is sustained or not.

“What often ends up happening is once the project kind of wraps up and we start taking our eye off it a little bit, the performance starts to fall back down again. And I think the answer why is because the culture hasn’t really changed all that much.”

(200)

#### 2. Strategies for normalizing new clinical innovations:

Participants outline numerous strategies to achieve the aforementioned indicators of effective normalization and facilitate the uptake of new clinical practices until they become default. Each highlighted normalization strategy was mapped to a category of implementation strategies and a specific ERIC implementation strategy when applicable.^20,27^ The thematic and concept mapping is outlined below and described fully in Table 2.

**a. The process to adopt and integrate novel clinical interventions into usual care is facilitated by a** patient approach **that starts slow and gains momentum over time.** This normalization strategy is related to the implementation category labeled ‘**Use Evaluative and Iterative Strategies**’, which was rated by an expert panel of implementation science and clinical experts as the most important and feasible cluster. Within this cluster of implementation strategies, interviewees emphasized a normalization approach that related to one ERIC strategy in particular: **‘Stage implementation scale up**’. According to participants, taking a slow and steady approach to normalizing new practices is critical to garnering initial support for the change.

“I took the gentle, slow approach. But I think we were more successful because of that…if we had charged in there and said, We want to in six months revamp the way we do safety’ it wasn’t gonna happen.”

(200)

After the local and slow uptake of the change, participants agreed that the practice often builds momentum and is normalized across the organization at increasing speed as the majority of staff are convinced of the practice’s utility.

“It became the norm, but it definitely takes time… you have that bell curve of when people adopt even practices that I wouldn’t say are new but are sort of maybe new on the radar. You get a critical mass. More like a tipping point…you continue to hope we can get traction over time, or that more evidence is found…those late adopters do eventually adopt.”

(199)

**b. The process to adopt and integrate novel clinical interventions into usual care is facilitated by strategies to gain** staff buy-in and ownership **over the practice change**. Participants described specific tactics leaders can employ to gain staff buy-in and ownership. One such strategy included engaging staff through conversations and surveys to solicit feedback and establish buy-in to ensure the change is the best fit. This aligns with multiple ERIC strategies that fall within the **‘Develop Stakeholder Interrelationships’** category: 1) ‘**Organize clinician implementation team meetings**’; 2) **‘Conduct local consensus discussions’**; and 3) **‘Use advisory boards and workgroups’**.

“The more that we can take input from bedside nurses and, there’d be times we would say okay so how do you think we could do this better? That wouldn’t be so burdensome from you? And if we could get buy-in from those people, I think that, that you had a better chance of things sticking.”

(206)

Another strategy discussed by participants to facilitate staff buy-in and promote change was providing ample education, information, and training around the practice, which maps to the **‘Train and Educate Stakeholders’** category and several ERIC strategies: **‘Conduct ongoing training’**; **‘Develop educational materials’**; **‘Distribute educational materials’**; and **‘Conduct educational meetings’**.

“We would continue to talk about it at division meetings, socialize it, in clinic, teach it to the residents, talk about it with the students. I think eventually most of those folks either started doing it or stopped grumbling about doing it. And then it became the norm.”

(199)

Lastly, building relationships and garnering support across departments made staff more willing to adopt a new practice in their own units if they saw early evidence of it working in other areas. This emerging theme aligns with the **‘Develop Stakeholder Interrelationships’** category and the **‘Build a coalition’**; **‘Identify early adopters’**; and **‘Capture and share local knowledge’** ERIC implementation strategies.

“When we presented on family-centered rounds to the exec committee and encouraged that this should be the standard of care for all pediatric services, the surgery service was excited to implement this…so it kind of cross-pollinates for people to talk about it in other settings.”

(125)

**c. The process to adopt and integrate novel clinical interventions into usual care is facilitated by using both** quantitative and qualitative indictors **for monitoring progress towards sustained change until dedicated monitoring is no longer needed.** This theme aligns with the **‘Audit and provide feedback’** and **‘Develop and organize quality monitoring systems’** ERIC implementation strategies, which fall under the **‘Use Evaluative and Iterative Strategies’** category.

Quantitative measures are important for auditing change for several reasons. First, auditing enables implementation leaders to track improvement with a greater degree of confidence than by relying on qualitative and anecdotal evidence alone. One participant described her institution’s efforts to reduce the amount of time it took for a patient to be transported from post-surgery to an inpatient unit:

“Continuing to monitor data and seeing that what we had put in place was being used, was being utilized was a helpful signal. And it’s been a few years now whether or not that continues to be the case is different. But I think for us, we celebrated when we on a monthly basis would gather around as the implementation team and look at the data and we could see that the people were using the tools that we had put in place…those kinds of metrics were important for us.”

(209)

Second, quantitative measurements track improvement on relevant quality-of-care, clinical, and performance outcomes that are linked to the practice change. Participants highlighted the value of tracking key measures of process and outcomes for both implementation leaders and relaying this clinical data back to providers to promote the use of the targeted innovation. This sub-theme aligns with the **‘Facilitate relay of clinical data to providers’** ERIC implementation strategy under the **‘Support Clinicians’** category.

“The other thing that’s important are the metrics behind it, especially for the clinical piece, reduction of safety events that are happening at our hospital. We did see ways that it was reducing errors. So that was a key metric. Like in an ideal setting, if we’re really practicing this mindset and it’s embedded, we’re going to see less safety events across the board.”

(202)

In addition to highlighting the importance of tracking quantitative measures, participants also identified crucial qualitative indicators, including positive feedback from staff and observable behavioral changes, especially without the need of reminders or “guardrails”. Another practice change example illustrated by a participant was changing the process of how a colonoscopy report is managed and integrated into the electronic health record to establish follow-up care and report quality measures:

“We saw that someone is initiating this, or someone is talking about it, or they’re already doing it. Or we hear the fellows are already doing these things. That’s how we know this sort of culture changes. When you use this anecdotal evidence that [staff] are initiating instead of just being passive.”

(208)

Ongoing monitoring and evaluation of both quantitative and qualitative outcomes facilitates adoption of a practice change, especially in early stages, by demonstrating leadership prioritization in the practice and holding staff accountable through reminders. This sub-theme aligns with the ‘**Remind clinicians’** ERIC implementation strategy under the **‘Support Clinicians’** category.

“When you’re trying to make a change, staff have to buy in, but you have to really find out and make sure that it’s actually happening…if you take your eye off the ball and you’re not following up periodically and saying, “Hey, how’s that going? Are we still doing it?” It doesn’t become part of your culture. It becomes a short-term change project and people lose interest.”

(201)

Despite the importance of tracking progress towards sustained change, participants emphasized that auditing is no longer needed when the change has truly been adopted as standard practice. At this point, the “guardrails” can be removed because the practice is truly integrated into behavioral and belief systems as the new norm.

“It wasn’t just something that was Meeting, I guess you should say and that everybody forgot about it the next month. You couldn’t, you didn’t have a choice, but to be involved in it because it was going to change the way that you took care of a patient. And now I can’t imagine going to a nurse who’s graduated last year and pulling it away from her ‘cause that’s all she knows now. It’s a part of our culture now.”

(203)

## DISCUSSION

This qualitative study investigated the critical issue of how clinical leaders gauge when a practice change becomes normalized and strategies they use to achieve this goal, addressing a substantial gap in the literature regarding facilitating and measuring the transformation of healthcare practices.^28^ The results of the study provide valuable insights into the indicators that signify when a novel clinical practice becomes normalized and the strategies employed to facilitate this transition.

The results highlight several key signals of when a new evidence-based practice, guideline, or approach becomes fully institutionalized into clinical care and have important differences from existing research on how to measure normalization of EBPs. The Sustainment Measurement System Scale is a 35-item scale designed to measure determinants and outcomes of efforts to sustain prevention programs and initiatives in community-based settings.^17,29,30^ Items investigate financial stability, responsiveness to community values, work done by coalitions/partnerships/networks, infrastructure, organizational capacity, implementation leadership, evaluation efforts, and sustainment outcomes. Conversely, issues of financial stability and funding sources did not reach thematic saturation in our qualitative interviews. Additionally, our results focused more on inner context buy-in and ownership to support normalization, while Palinkas et al.,^17^ identified coalitions, partnerships, and networks as important factors to establishing a commitment to the continued operation of a change effort. Similar to the Sustainment Measurement System Scale,^17^ we identified ongoing monitoring efforts as a critical signal of normalization/sustainment.

Our findings are consistent with previous research on the implementation and sustainment of new clinical practices, while also adding to the literature gap on how an innovation becomes routinized.^31–33^ Previous research has shown that integration into existing workflows, scaling across the entire organization, and strong staff buy-in and ownership are all important factors for successful implementation of new clinical practices.^23–25^ All of these strategies require that clinical leaders are involved in every stage of implementation to conduct initiating, monitoring, and sustaining change. They play a valuable role with allocating resources, problem solving, scaling the practice change, and engaging frontline staff.^34–36^ The critical need to engage staff to garner buy-in and ownership is emphasized as an effective implementation strategy in the literature and validated in this study.^37^ Our findings add to the existing literature on the topic of staff buy-in is that,^38–40^ in addition to an implementation strategy, staff ownership is also an important indication that a practice change has been internalized and normalized among staff. For a change to become integrated in practice, it requires staff to understand and believe in the value the change, for both the staff and patient benefit.

Additionally, our findings add additional support to evidence that the process of initiating practice change from early planning to normalization is not a direct path.^41,42^ Instead, our study supports existing evidence that the change process requires staff involvement, feedback, monitoring and revision before successful integration occurs.^34,43,44^ For a change to become integrated in practice, it goes through iterative stages outlined in Fig. 1 to transform from the unfamiliar to the familiar, from novel to routine. These findings align with previous research including Brewster et al.,^34^ Yin et al.,^45^ and Rogers’ diffusion of innovation model,^46^ which describe this staged approach of integrating a new practice. Our findings further call attention to why sustaining new practice changes are difficult, and often not successful, because of the required planning and time it requires to allocate resources and align changes with staff attitudes^1,13^; however, our in-depth interviews offer a glimpse into how practice change strategies can aid in success.

### Strengths and limitations

Study findings should be interpreted in light of methodological strengths and limitations. Our study has several strengths. First, we conducted in-depth interviews with clinical leaders from a variety of healthcare settings across the US. This allowed us to collect rich data on how diverse clinical leaders perceive and achieve practice change. Second, we used a rigorous data analysis process grounded in theory and a consensual qualitative analytic approach, to ensure the validity and reliability of our findings. Specifically, our application of the established ERIC implementation strategies during data analysis helped to ensure conceptual clarity, consistency, and relevance of our findings to both the field of implementation science and clinical practice.

Our study also has some limitations. First, our sample size was relatively small and lacked diversity. Participants were predominantly highly educated females, thereby limiting the representativeness and generalizability of our findings. Another weakness was that we did not collect data on race/ethnicity. Additionally, the sample was limited to clinical leaders in implementation or quality improvement and did not include floor staff. While clinical leader perspectives are important, the perspectives of frontline staff actively involved with patients and day-to-day care are necessary for a holistic understanding on how and when a new evidence-based practice becomes standardized. Despite these limitations, our study provides valuable insights into how, when, and why an evidence-based clinical practice transitions from new to normal.

### Implications

Our findings can be used by researchers, clinicians, and healthcare administrators to develop and utilize effective strategies for achieving the normalization of new clinical practices. Specifically, a high yield approach for healthcare implementation teams seeking to integrate new clinical practices would be to test the ERIC implementation strategies that aligned with methods identified by interviewees. Clinicians should be aware of the indicators and strategies that can facilitate the normalization of new clinical practices in clinical care. By integrating new practices into existing workflows, scaling them across the entire organizational unit, and gaining staff buy-in and ownership, clinicians can help to ensure that new practices are implemented successfully and sustained over time. Healthcare administrators should provide clinicians with the necessary support and resources to implement new clinical practices effectively. This includes providing training and education, involving staff in the planning and implementation process, and recognizing and rewarding clinicians for using new practices. Policy decision-makers can support the normalization of evidence-based practices in clinical care by funding implementation research in these settings, financing the development of tools and resources to support clinicians, and creating and aligning incentives for clinicians and healthcare organizations to adopt new practices.

### Future research

Future research should be conducted to further explore the factors that influence the normalization of new clinical practices in clinical care and develop instruments that implementation leaders can use to systematically measure the clinical change process based on the indicators identified in this study. Specifically, these findings should be applied to design a transferable qualitative interview guide that can be used by researchers and practitioners seeking to monitor and evaluate the progression of an EPB to normalization. Though development of an interview guide is outside the scope of this study, major themes should inform future questions, such as: 1) How is the EBP integrated into existing workflows? 2) What is the level of staff buy-in and ownership over the EBP? 3) To what extent is the EBP continuously implemented without requiring ongoing monitoring? Additionally, future research could involve the conduct of studies testing the implementation strategies identified in this study to explore their effectiveness and ability to be scaled on a broader scale. Other research could examine best practices for measuring and tracking the progress of implementing new clinical practices and explore how clinical leaders can create a culture of continuous improvement and sustainability within their organizations, as well as implementation strategies that can best support frontline staff respond to lead and carry out practice change.

## CONCLUSION

In summary, this study provides a comprehensive examination of how, when, and why a clinical practice change becomes normalized within healthcare organizations. The findings emphasize the importance of indicators that signify the transition and the strategies that can facilitate this process. Understanding these indicators and strategies is crucial for healthcare leaders, as it enables them to better navigate the complexities of practice change and ensures the efficient and effective integration of evidence-based practices into standard care. Ultimately, the study contributes valuable insights to the broader field of healthcare implementation and offers a roadmap for successful clinical practice change.

## Figures and Tables

**Table 1:** Participant and Setting Characteristics

*Participant Characteristic*	N = 17
Median Age Range	46–55 years
Mean number of years in healthcare	22 years
Graduate degree attainment	15
Sex	
Female	14
Male	2
Not reported	1
*Setting Characteristics*	N = 17
Institution type	
Academic	14
Non-academic	2
Community health center	1
In-patient size	
Small	1
Medium	5
Large	10
Not applicable	1
Region	
Northeast	7
Southeast	3
South	1
Midwest	4
West	1
Southwest	1

**Table 2:** Qualitative themes mapped to ERIC Implementation Strategies and Categories

Qualitative Theme	Implementation Category^1^	ERIC Implementation Strategy^2^	Definition^2^
*The process to adopt and integrate novel clinical interventions into usual care is facilitated by a patient approach that starts slow and gains momentum over time*.	Use Evaluative and Iterative Strategies	Stage implementation scale up	Phasing implementation efforts by starting with small pilots or demonstration projects and gradually move to a system wide rollout.
*The process to adopt and integrate novel clinical interventions into usual care is facilitated by strategies to gain staff buy-in and ownership over the practice change*.			
Sub-theme: An identified strategy to gain staff buy-in and ownership included engaging staff through conversations and surveys.	Develop Stakeholder Interrelationships	Organize clinician implementation team meetings	Develop and support teams of clinicians who are implementing the innovation and give them protected time to reflect on the implementation effort, share lessons learned, and support one another’s learning.
		Conduct local consensus discussions	Include local providers and other stakeholders in discussions that address whether the chosen problem is important and whether the clinical innovation to address it is appropriate.
		Use advisory boards and workgroups	Create and engage a formal group of multiple kinds of stakeholders to provide input and advice on implementation efforts and to elicit recommendations for improvements.
Sub-theme: Another identified strategy to facilitate staff buy-in was through education.	Train and Educate Stakeholders	Conduct ongoing training	Plan for and conduct training in the clinical innovation in an ongoing way.
		Develop educational materials	Develop and format manuals, toolkits, and other supporting materials in ways that make it easier for stakeholders to learn about the innovation and for clinicians to learn how to deliver the clinical innovation.
		Distribute educational materials	Distribute educational materials (including guidelines, manuals, and toolkits) in person, by mail, and/or electronically.
		Conduct educational meetings	Hold meetings targeted toward different stakeholder groups (e.g., providers, administrators, other organizational stakeholders, and community, patient/consumer, and family stakeholders) to teach them about the clinical innovation.
Sub-theme: Building relationships and garnering support across departments made staff more willing to adopt a new practice in their own units	Develop Stakeholder Interrelationships	Build a coalition	Recruit and cultivate relationships with partners in the implementation effort.
		Identify early adopters	Identify early adopters at the local site to learn from their experiences with the practice innovation.
		Capture and share local knowledge	Capture local knowledge from implementation sites on how implementers and clinicians made something work in their setting and then share it with other sites.
*The process to adopt and integrate novel clinical interventions into usual care is facilitated by using both quantitative and qualitative indictors for monitoring progress towards sustained change*.	Use Evaluative and Iterative Strategies	Audit and provide feedback	Collect and summarize clinical performance data over a specified time period and give it to clinicians and administrators to monitor, evaluate, and modify provider behavior.
		Develop and organize quality monitoring systems	Develop and organize systems and procedures that monitor clinical processes and/or outcomes for the purpose of quality assurance and improvement.
Sub-theme: Tracking key quantitative measures of process and outcomes and relaying this clinical data back to providers promotes the use of the targeted innovation.	Support Clinicians	Facilitate relay of clinical data to providers	Provide as close to real-time data as possible about key measures of process/outcomes using integrated modes/channels of communication in a way that promotes use of the targeted innovation.
Sub-theme: Ongoing monitoring and evaluation facilitates adoption of a practice change, especially in early stages, by demonstrating leadership prioritization in the practice and holding staff accountable through reminders	Support Clinicians	Remind clinicians	Develop reminder systems designed to help clinicians to recall information and/or prompt them to use the clinical innovation.

1. Waltz, T.J., Powell, B.J., Matthieu, M.M. et al. Use of concept mapping to characterize relationships among implementation strategies and assess their feasibility and importance: results from the Expert Recommendations for Implementing Change (ERIC) study. Implementation Sci 10, 109 (2015). https://doi.org/10.1186/s13012-015-0295-0

2. Powell, B.J., Waltz, T.J., Chinman, M.J. et al. A refined compilation of implementation strategies: results from the Expert Recommendations for Implementing Change (ERIC) project. Implementation Sci 10, 21 (2015). https://doi.org/10.1186/s13012-015-0209-1

## Data Availability

The datasets used and/or analyzed during the current study are not publicly available due to privacy and confidentiality of our research participants, but are available from the corresponding author on reasonable request.
